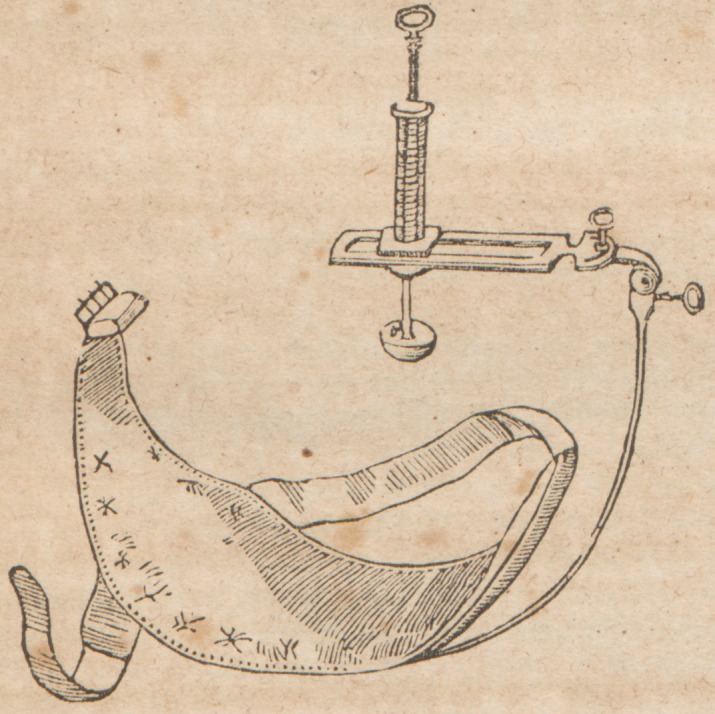# Editorial and Miscellaneous

**Published:** 1864-03

**Authors:** 


### C. S. Medical & Surgical Journal.

RICHMOND, MARCH, 1864.

*AYRES & WADE......PUBLISHERS AND PROPRIETORS.*

### *Treatment of Aneurism by Compression.*

Every attempt to cure aneurism rests upon this principle—to obliterate the artery at the seat of disease, and turn the current of blood into other and more circuitous routes.

Imitating nature in its spontaneous method of cure, by the gradual deposit of fibrine in the aneurism and the ultimate occlusion of the vessel, we have the brilliant conception of Hunter, who ligates the artery between the heart and the sac; the ingenious method of Brasdor, producing a reflex stasis in the tumor by tying the distal portion of the artery, and the direct, but hazardous effort to extirpate the tumor by laying open the sac, and ligating both ends of the vessel.

The attempt to cure this formidable surgical condition by compression, whether direct or indirect—originating probably with the French, but first put into successful operation by the Irish surgeons—covers the same idea. The circulating fluid is prevented from passing along the dilated or ruptured tube with its usual velocity and momentum, and is thus encouraged to deposit fibrinous layers, which eventually occlude the calibre of the tube. This procedure holds this great advantage over the process by ligature, for it avoids the necessity of a surgical and dangerous operation, and leaves the parts approximating as closely as possible to a natural state.

The difficulties in this mode of treatment are, first, how to compress the artery, without at the same time checking the circulation of blood in the whole limb; secondly, so to graduate the compression as that a certain amount of blood may continue to pass through the tube to furnish the supply of fibrine required to effect the cure; and third, to prevent the intense pain and other more serious consequences, where too much force is exerted at the point of pressure.

The instrument delineated in the accompanying engraving, the invention of Mr. Earnest Hart, of Marylebone Hospital, overcomes, in a great degree, the impediments to success, and is so simple as scarcely to require an explanation. It is applied by a broad strap to the body, and the limb is thus entirely free from general pressure. The compressor is turned on its pivot and adjusted over the point of selection, and the amount of pressure is then registered by the action of the spiral spring upon which the pad rests, and a dial plate gives, at a glance, the number of pounds' weight thrown upon the artery at any time.

Such an instrument as this offers all the advantages of *digital* compression, heretofore the most successful mode of applying this principle to the cure of aneurismal tumors, and is always to be relied on after once carefully applied; whilst, without numerous relays of patient and careful assistants, the digital method will result in failure.

It may be also remarked that, by registering accurately the amount of pressure required to produce the desired effect, we may at last approximate closely to the probable number of pounds' weight necessary to obtain the object, in an average number of cases.

Mr. Hart deserves also to be commended for his ingenious application of compression in popliteal aneurism, by flexing the leg on the thigh, and bandaging the whole limb firmly, thus obliterating the sac by a direct pressure. The English journals report twelve successful cases since 1858, when the idea was first promulgated by its author.

---

*Association to Purchase Artificial Limbs for Maimed Soldiers.*

We notice, with pleasure, the successful organization in this city of the association to obtain artificial limbs for our brave soldiers who have been mutilated in the defence of liberty and honor. The happy idea was originated by the Rev. Dr. Marshall, of Mississippi, who was nobly supported by many public-spirited persons, so that, in a very few days, not less than fifty thousand dollars were collected.

It is creditable to the ingenuity of our people to say that, spite of many difficulties, it is probable that a sufficient number of good and serviceable legs can be manufactured in the Confederacy. The society is now in successful operation. The president, Dr. Marshall, with a central directory, has made some progress in contracting for artificial limbs, whilst the treasurer, Wm. H. Macfarland, Esq., is ready to receive contributions from those who are disposed to aid in a praiseworthy object. Maimed soldiers, desiring to obtain artificial limbs, will apply to Medical Director Carrington, Corresponding Secretary, who will give due attention to their claims.

## Figures and Tables

**Figure f1:**